# Use of non‐vitamin K antagonist oral anticoagulants in Colombia: A descriptive study using a national administrative healthcare database

**DOI:** 10.1002/pds.5124

**Published:** 2020-10-15

**Authors:** Jorge E. Machado‐Alba, Andrés Gaviria‐Mendoza, Manuel E. Machado‐Duque, Carlos Tovar‐Yepes, Ana Ruigómez, Luis Alberto García Rodríguez

**Affiliations:** ^1^ Grupo de Investigación en Farmacoepidemiología y Farmacovigilancia Universidad Tecnológica de Pereira–Audifarma S.A. Pereira Colombia; ^2^ Grupo Biomedicina, Fundación Universitaria Autónoma de las Américas Pereira Colombia; ^3^ Centro Español de Investigación Farmacoepidemiológica (CEIFE) Madrid Spain

**Keywords:** anticoagulation, drug utilization, nonvalvular atrial fibrillation, non‐vitamin K antagonist oral anticoagulants, observational study, pharmacoepidemiology

## Abstract

**Purpose:**

We aimed to describe time‐trends in the use of NOACs among a group of ambulatory patients with nonvalvular atrial fibrillation (NVAF) in Colombia and to describe treatment patterns and user characteristics.

**Methods:**

Using the Audifarma S.A administrative healthcare database in Colombia, we identified 10 528 patients with NVAF aged at least 18 years between July 2009 and June 2017 with a first prescription (index date) for apixaban, dabigatran or rivaroxaban (index NOAC) and followed them for at least year (max, 8.0 years, mean 2.2 years). We described patient characteristics, NOAC use over time, and the dose of the first NOAC prescription.

**Results:**

A total of 2153 (20.5%) patients started on apixaban, 3089 (29.3%) on dabigatran and 5286 (50.2%) on rivaroxaban. The incidence of new users of apixaban and rivaroxaban increased over study years while for dabigatran it decreased. Mean age at the index date was: 78.5 years (apixaban), 76.5 years (dabigatran), 76.0 years (rivaroxaban). The percentage of patients started NOAC therapy on the standard dose was: apixaban 38.0%, dabigatran 30.9%, rivaroxaban 56.9%. The percentage still prescribed their index NOAC at 6 months was apixaban 44.6%, dabigatran 51.4%, rivaroxaban 52.7%. Hypertension was the most common comorbidity (>80% in each NOAC cohort).

**Conclusion:**

During the last decade, the incidence of NOAC use in patients with NVAF affiliated with a private healthcare regime in Colombia has markedly increased. Future studies should evaluate whether the large number of patients with NVAF starting NOAC treatment on a reduced dose are done so appropriately.

**KEY POINTS**

- The marked increase in the use of rivaroxaban and apixaban to patients with NVAF affiliated with a private healthcare regime in Colombia over the last decade indicates growing confidence in the prescribing of these two NOACs among physicians in the country.
- As substantial numbers of patients with NVAF affiliated with a private healthcare regime in Colombia appear to be prescribed a reduced dose NOAC, studies are now warranted to evaluate the extent to which this is done appropriately—in accordance with the drug labels.

**Abbreviations**
ACE: angiotensin‐converting‐enzyme
AF: atrial fibrillation
H_2_RAs: H2‐receptor antagonists
ICD: international classification of diseases
NOAC: non‐vitamin K antagonist oral anticoagulant
NSAIDs: nonsteroidal anti‐inflammatory‐inflammatory drugs
NVAF: nonvalvular atrial fibrillation
OAC: oral anticoagulant
PPIs: proton pump inhibitors
VTE: venous thromboembolism

## 1 INTRODUCTION

Atrial fibrillation (AF) is a common cardiac arrhythmia with a prevalence that increases with age.^1^ It is estimated that one in four middle‐aged adults in Europe and the United States will develop AF in their lifetime.^2^ The arrhythmia is associated with a 4‐ to 5‐fold increase in the risk of ischaemic stroke^3^ and a 1.5‐ to 2‐fold increased risk of all‐cause mortality.^2^ Most epidemiological data on AF have come from Western populations; however, analysis of national healthcare databases show that AF also represents a substantial public health burden in Latin America, with estimated country‐specific prevalences of around 13% among individuals aged 70 years or more.^4^ Moreover, evidence suggests that the prevalence of AF, stroke, and associated mortality has increased dramatically in Latin America, likely due to the combined effect of the aging population and poor control of major risk factors such as hypertension.^5^ However, little is known about the management of patients with AF in this area of the world since the introduction of non‐vitamin K antagonist oral anticoagulants (NOACs) as an alternative option for stroke prophylaxis in this patient population in the last decade. Non‐vitamin K antagonist oral anticoagulants have been shown to be noninferior to warfarin in reducing the risk of stroke and systemic embolism in patients with AF, and to have a superior safety profile. This has been shown in the overall pivotal clinical trial populations in which their approval was based,^6^, ^7^, ^8^ as well as in subanalyzes of these trials restricted to participants in Latin America.^9^ Unlike warfarin, the fixed dose regimens and predictable pharmacokinetics of NOACs mean that regular clinic visits for coagulation monitoring are not needed, and this may be especially beneficial in regions of Latin America where access to effective monitoring services may be difficult. Most data on the use of NOACs have come from American or European populations, and data from Latin America are lacking. Using a national administrative healthcare database, we aimed to describe time‐trends in the use of NOACs among ambulatory patients with nonvalvular atrial fibrillation (NVAF) in Colombia, and characteristics of patient users and of the specific NOAC prescribed.

## 2 MATERIALS AND METHODS

### 2.1 Study design and data source

A cohort study was conducted using data from the Audifarma S.A administrative healthcare database, the largest drug dispensing company in Colombia with information from patients at all levels of care. The Colombian Health System offers universal health coverage through two regimes—the contributory regime that is paid by the employer and the worker, and a subsidized regime paid by government free of charge. The Audifarma S.A database contains patient information for 4.8 million people affiliated to the contributory regime of five private Colombian health insurance companies, and it has been validated in multiple studies on medication use in the Colombian population. The database uses the Business Objects tool on a platform in Oracle where all daily drug dispensations to patients affiliated with insurers are stored. The date of medication dispensation, quantity, dose, route of drug administration, and indication for the prescription are all recorded. Information held also includes sociodemographic variables for the patient, and diagnoses using the International Classification of Diseases Version 10 (ICD‐10). While both regimes enable warfarin to be prescribed for anticoagulation purposes, the prescribing of NOACs requires a request through a technological tool called MIPRES. The Colombian Atrial Fibrillation Treatment Guidelines state that either warfarin or a NOAC can be prescribed for the prevention of embolic events.^10^ The Audifarma database has been validated in multiple studies that show how medications are used in the Colombian population.^11^, ^12^, ^13^ The source population included patients in the contributory regime aged at least 18 years between July 2009 and June 2017 with at least 1 year of enrollment with their insurance provider and at least 1 year of available data following their first recorded outpatient health contact to guarantee a certain level of continuity with health services. No patient identifying information was used in this study. The study protocol was approved by the bioethics committee of the Universidad Tecnológica de Pereira, Colombia.

### 2.2 NOAC study cohorts

From within the source population, three mutually exclusive cohorts of first‐time users of NOACs (apixaban, dabigatran or rivaroxaban) were identified with the date of first NOAC prescription (index NOAC) set as the index date. If a patient had a prescription for another anticoagulant (eg, warfarin) in the year before their index date they were classified as non‐naïve, while patients with no prescription for another anticoagulant in the year before their index date were classed as naïve. We excluded patients who were prescribed two different NOACs on the same day. Patients who qualified as a first‐time user of more than one NOAC at different times during the study period (ie, switchers) were assigned to the cohort of the first prescribed NOAC. For each NOAC cohort we subsequently only retained patients with a record of AF (ICD‐10 code I48) before the index date or in the 2 weeks after the index date. Patients with a record of mitral stenosis (ICD‐10 codes: I050, I05X, I052), valvular replacement (ICD‐10 codes: Z952‐Z954), and others stenosis (ICD‐10 codes: I058, I059, I080, I081, I083, I088) during this time interval were excluded to identify only those patients with NVAF because there are no specific ICD‐10 codes for NVAF. All patients were followed up for at least 1 year from the index date, until leaving the health plan, death or end of study data collection (December 2017).

### 2.3 Study outcomes and covariates

#### 2.3.1 Characteristics of first‐time NOAC users with NVAF

We extracted data on patient demographics (age and sex), comorbidities in the year before the index date including cardiovascular comorbidities (myocardial infarction, heart failure, ischaemic stroke, haemorrhagic stroke, venous thromboembolism (VTE) and hypertension) and other comorbidities (diabetes mellitus, chronic obstructive pulmonary disease, gastrointestinal bleeding, severe renal failure, and cancer). We also extracted data on the following medications prescribed in the year before the index date: other anticoagulants including warfarin and low‐molecular‐weight heparin (LMWH), antiplatelets (low‐dose aspirin and clopidogrel), antiarrhythmic drugs, antihypertensive drugs, statins, antidiabetic drugs, nonsteroidal anti‐inflammatory drugs, acid‐suppressive drugs, antidepressants and oral steroids (see Table S1). Polypharmacy was assessed as the number of different medications prescribed in the 2 months before the index date. We also identified patients with a prescription for another anticoagulant drug (including warfarin and low‐molecular weight heparin) at any time before the index date, and classed these patients as anticoagulant non‐naïve; all other patients were classed as anticoagulant naïve.

#### 2.3.2 Characteristics of the index NOAC prescription

For the index NOAC prescription and for subsequent NOAC prescriptions, we extracted information on the number of pills prescribed, the dose and the posology, and estimated the duration of each prescription using these instructions. If there was missing information on the daily dose, we made an assumption about the most likely dose based on assessment of the timing of subsequent prescriptions to that patient. We assessed the dose of the index NOAC prescription as well as the dose prescribed 3 months later. For all patients, we calculated the duration of the first episode of continuous NOAC treatment. Continuous treatment was when there was either no gap in treatment of >30 days between the end of the supply of a prescription and the start of the next prescription for the same NOAC, or no further prescription after the end of the previous one.

### 2.4 Statistical analysis

For each NOAC cohort, the characteristics of patients and of the index NOAC prescription (at the start of follow‐up and at 3 and 6 months) were described using frequency counts and percentages for categorical data, and means with SD for age. To evaluate trends in NOAC prescribing for stroke prevention in AF over time, the number of patients with NVAF newly prescribed a NOAC was described for each study year. In the calculation of incidence rates, we used only patients enrolled with two of the five healthcare providers contributing to the database (Salud Total and Compensar, corresponding to 3.6 of the 4.8 million patients in this study) for the numerator. This was because the denominator for this calculation—the total number of patients in the database (the exact number of individuals affiliated with the insurance regime in each year)—was only available for these two providers. Incidence rates of new users of NOACs with NVAF were calculated for each study year and were expressed per 10 000 individuals. We also calculated the percentage use for each OAC dispensed out of all OACs for each study year. In an analysis comparing the proportions of use of the two insurance companies vs the other three, in which the exact number of the total membership was not known, it was found that the proportions of use of NOACs was the same. All data analysis was conducted using SPSS Statistics Version 25 (IBM) for Windows.

## 3 RESULTS

**FIGURE 1:**
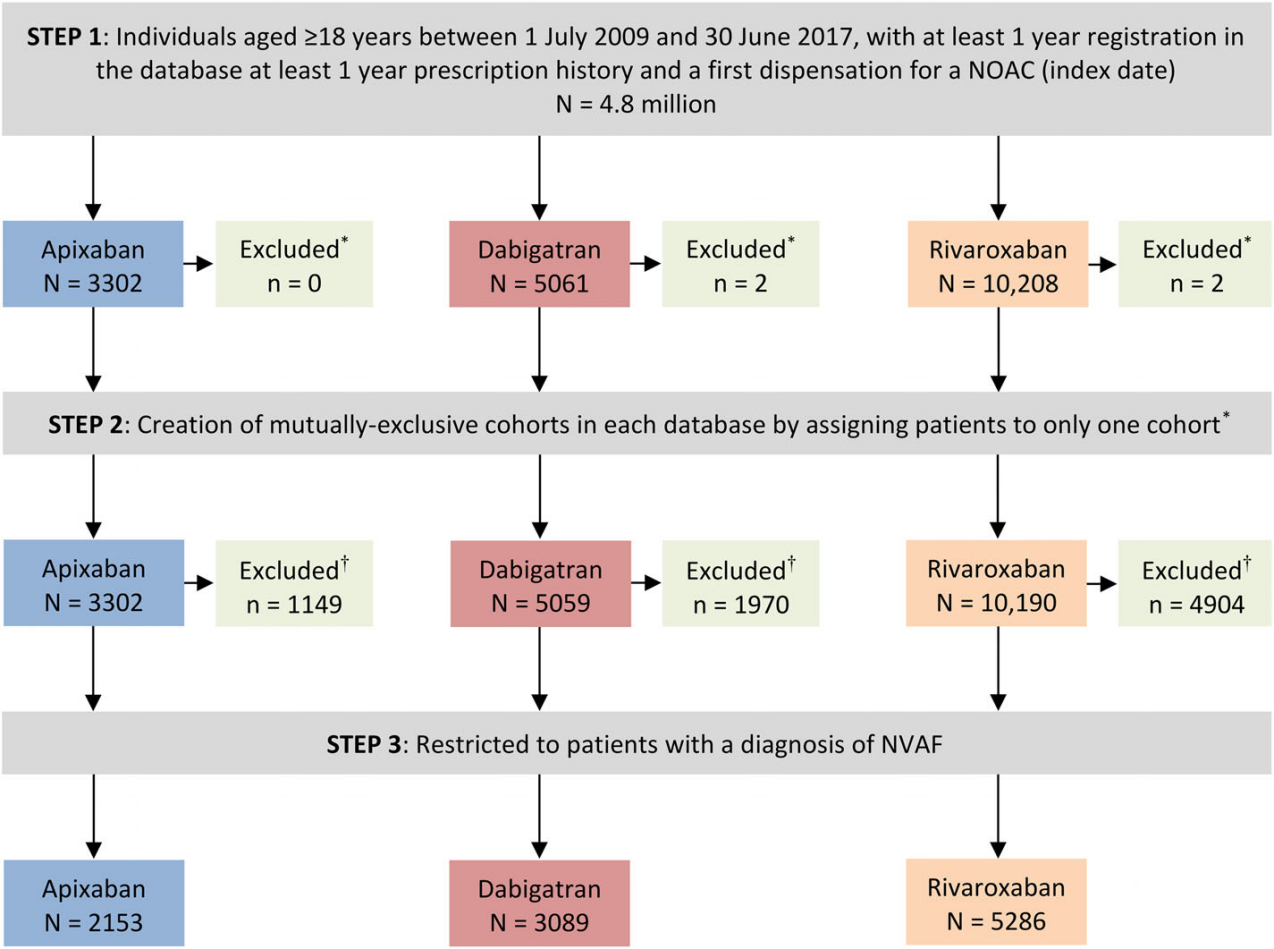
Flowchart depicting the identification of the three NOAC study cohorts. *Patients dispensed two different NOACs on the same date. †Patients were excluded if they had a code for mitral stenosis/valvular replacement before the index date or in the 2 weeks after the index date, or if they had a NOAC dispensation with no associated diagnosis. NOAC, non‐vitamin K antagonist oral anticoagulant; NVAF, nonvalvular atrial fibrillation [Colour figure can be viewed at wileyonlinelibrary.com]

**FIGURE 2:**
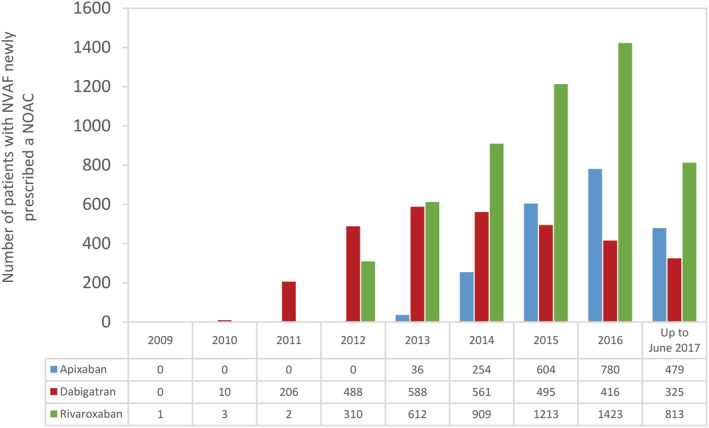
Number of first‐time users of apixaban, dabigatran and rivaroxaban with NVAF by study year (July 2009 to June 2017). Data are from two clinical benefit providers (Salud Total and Compensar). NOAC, non‐vitamin K antagonist oral anticoagulant; NVAF, nonvalvular atrial fibrillation [Colour figure can be viewed at wileyonlinelibrary.com]

**FIGURE 3:**
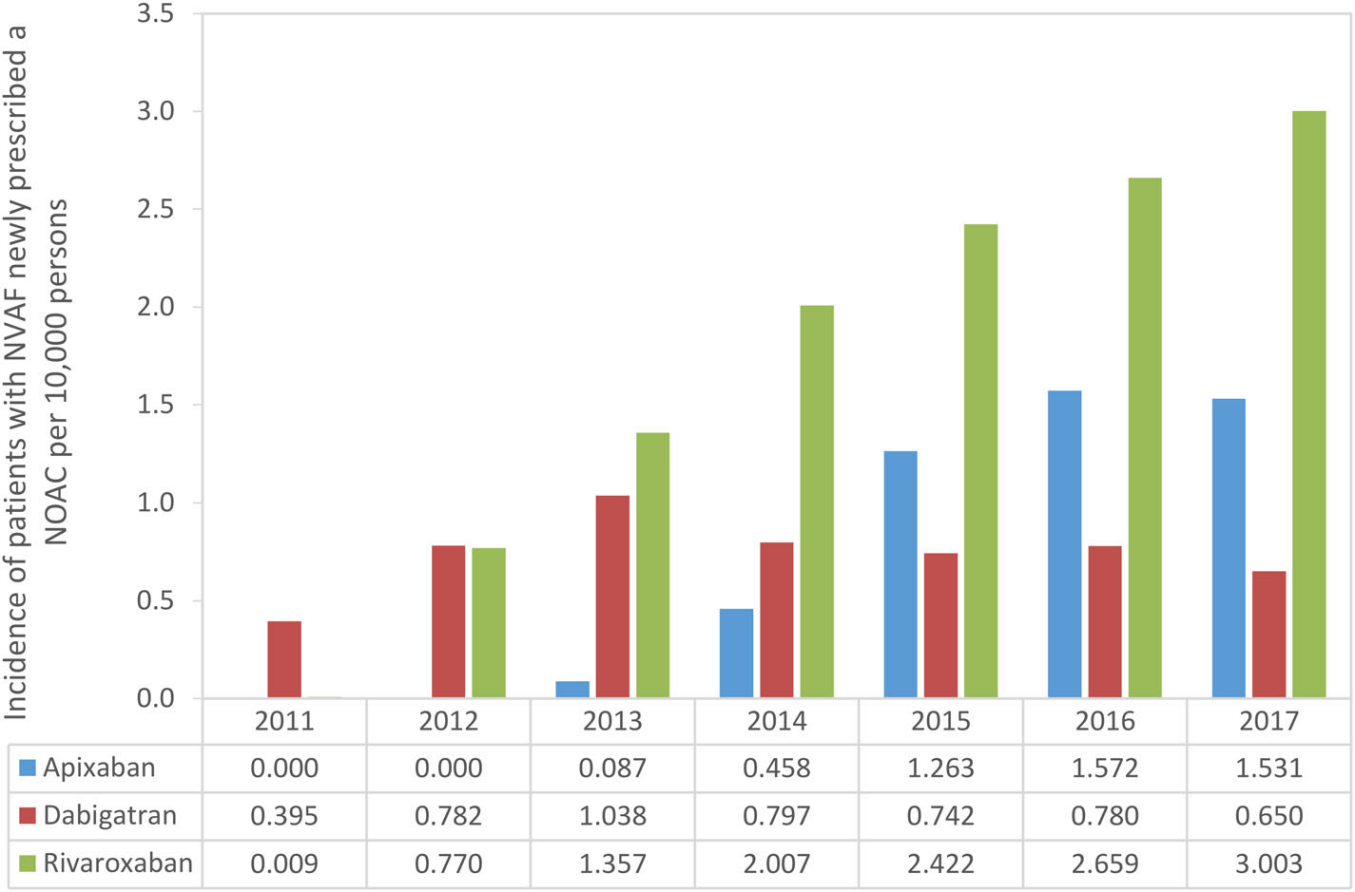
Incidence of patients with NVAF newly prescribed a NOAC per 10 000 persons, by study year. Data for 2017 were from January to December inclusive. Data are from two clinical benefit providers (Salud Total and Compensar). NOAC, non‐vitamin K antagonist oral anticoagulant; NVAF, nonvalvular atrial fibrillation [Colour figure can be viewed at wileyonlinelibrary.com]

**FIGURE 4:**
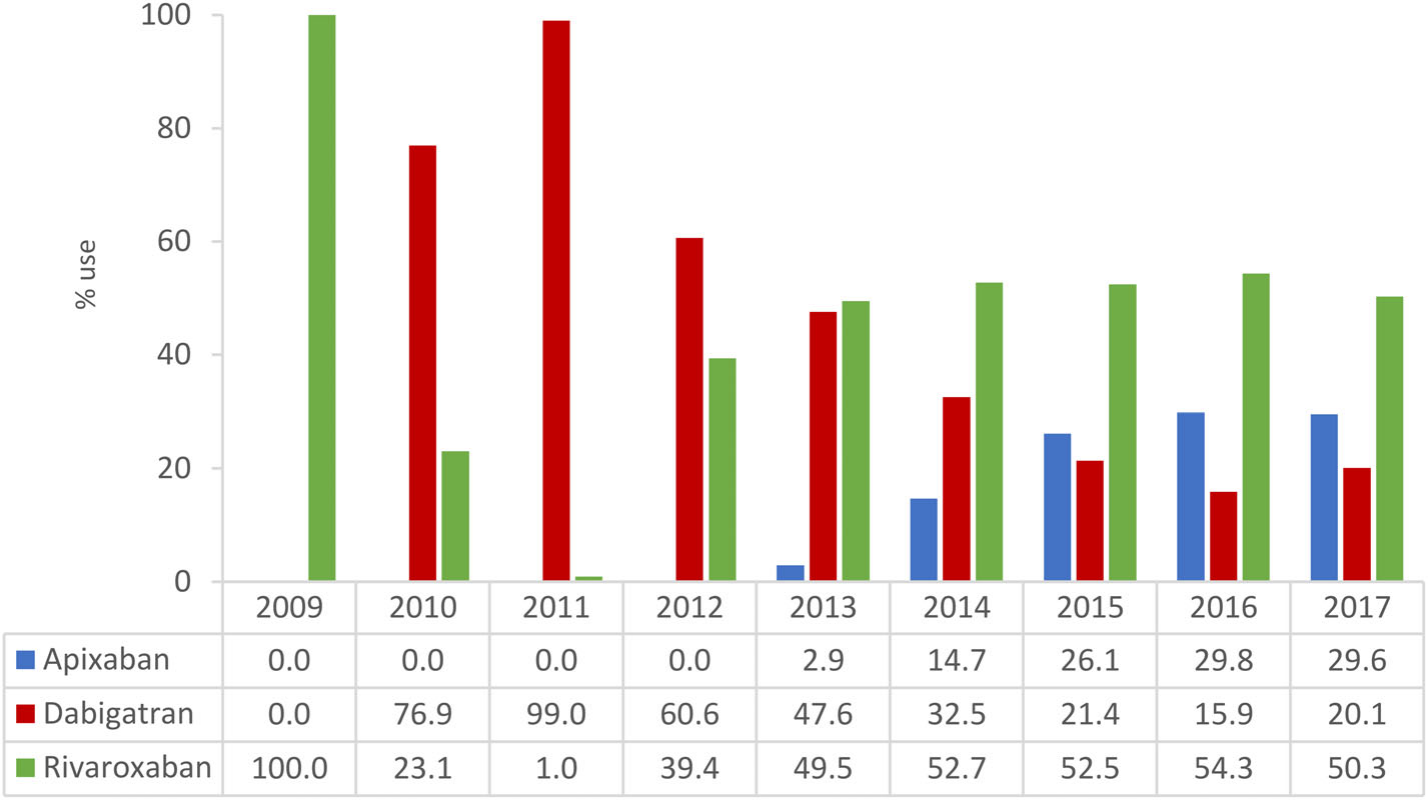
Percentage individual NOAC use among patients with NVAF by study year. Data are from two clinical benefit providers (Salud Total and Compensar). NOAC, non‐vitamin K antagonist oral anticoagulant; NVAF, nonvalvular atrial fibrillation [Colour figure can be viewed at wileyonlinelibrary.com]

A flowchart depicting the identification of the three NOAC cohorts is shown in Figure 1. Among a source population of 4.8 million individuals in the Audifarma database meeting the study inclusion criteria, we identified 10 528 patients with NVAF who were new users of a NOAC: 2153 (20.5%) started on apixaban, 3089 (29.3%) on dabigatran and 5286 (50.2%) on rivaroxaban. As shown in Figure 2, the number of patients with NVAF starting NOAC therapy on apixaban (36 in 2013, 780 in 2016) or rivaroxaban (310 in 2012, 1423 in 2016) increased markedly over study years, while the number of patients with NVAF starting NOAC therapy on dabigatran (488 in 2012, 416 in 2016) decreased (in Colombia, dabigatran was approved in 2008, rivaroxaban was approved in 2011, and apixaban was approved in 2013). These time‐trends are reflected in Figure 3, which shows incidence rates of patients with NVAF newly prescribed one of the three different NOACs, and also in Figure 4, which shows percentage use in each study year. The mean follow‐up was 2.2 years (minimum 1 year; maximum, 8 years). Approximately half of patients were still prescribed their index NOAC at 6 months (apixaban 44.6%, dabigatran 51.4% and rivaroxaban 52.7%).

### 3.1 Characteristics of first‐time users of a NOAC with NVAF

Characteristics of the three study cohorts are shown in Tables 1 and 2. There were slightly more males than females in each cohort and the mean age was similar between cohorts, albeit slightly higher among apixaban users (apixaban 78.5 years, dabigatran 76.5 years, and rivaroxaban 76.0 years). Approximately two‐thirds of patients were anticoagulant naïve (apixaban 70.5%, dabigatran 64.7% and rivaroxaban 65.8%). The percentage of anticoagulant naïve patients with NVAF starting NOAC therapy was fairly constant across the study period: 54.7% in 2012 and 57.1% in 2016. Hypertension was recorded in the vast majority (>80%) of patients, heart failure in about one third of patients and diabetes mellitus in about one fifth of patients, while approximately 1 in 7 patients had a record of severe renal failure (mean age of these patients was 80.7 years). A history of VTE was slightly more frequent among patients starting on rivaroxaban (6.3%) compared with those starting on apixaban (2.5%) or dabigatran (3.5%). Among all new users of NOACs with NVAF, 58.4% had been prescribed more than five different medications in the 2 months before starting NOAC therapy, and 25.3% had been prescribed at least 10 different medications in this time period. The most frequently prescribed medications in the year before starting NOAC therapy were beta‐blockers (59.1%), statins (55.8%), low‐dose aspirin (44.3%), proton pump inhibitors (44.3%), and diuretics (41.8%). Additionally, it should be noted that 10.6% used clopidogrel. During 12 months of follow‐up, no patients received a NOAC and LMWH concomitantly, but 3363 patients (31.9%) received a NOAC and an antiplatelet agent (all low‐dose aspirin) concomitantly.

**TABLE 1:** Characteristics of patients with NVAF prescribed a NOAC (July 2009 to June 2017)

Characteristic	Apixaban	Dabigatran	Rivaroxaban
	N = 2153	N = 3089	N = 5286
Mean age (SD), y	78.5 (11.0)	76.5 (11.0)	76.0 (11.0)
Males	1206 (56.0)	1696 (54.9)	3117 (59.0)
Anticoagulant naïve	1517 (70.5)	1999 (64.7)	3480 (65.8)
Number of PCP visits			
<3	546 (25.4)	829 (26.8)	1280 (24.2)
4‐9	303 (14.1)	496 (16.1)	780 (14.8)
10‐19	708 (32.9)	1002 (32.4)	1768 (33.4)
20‐29	338 (15.7)	459 (14.9)	855 (16.2)
≥30	258 (12.0)	303 (9.8)	603 (11.4)
Cardiovascular comorbidities^a^			
Hypertension	1724 (80.1)	2533 (82.0)	4359 (82.5)
Ischaemic stroke	150 (7.0)	300 (9.7)	395 (7.5)
Myocardial infarction	96 (4.5)	119 (3.9)	274 (5.2)
Heart failure	683 (31.7)	1115 (36.1)	2010 (38.0)
VTE	55 (2.5)	107 (3.5)	334 (6.3)
Haemorrhagic stroke	9 (0.4)	20 (0.6)	33 (0.6)
Other comorbidities^b^			
COPD	320 (14.9)	456 (14.8)	870 (16.5)
Diabetes mellitus	432 (20.1)	710 (23.0)	1161 (22.0)
Gastrointestinal bleeding	81 (3.8)	25 (0.8)	195 (3.7)
Severe renal failure	350 (16.3)	452 (14.6)	831 (15.7)
Cancer	138 (6.4)	190 (6.2)	347 (6.6)

Note: Data are n (%) unless otherwise specified.

Abbreviations: COPD, chronic obstructive pulmonary disease; NOAC, non‐vitamin K antagonist oral anticoagulant; NVAF, nonvalvular atrial fibrillation; PCP, primary care practitioner; VTE, venous thromboembolism.

^a^ In the year before the index date.

^b^ On the index date or in the year before the index date.

**TABLE 2:** Medication use in the year before the start of NOAC therapy in patients with NVAF (July 2009 to June 2017)

Medication	Apixaban	Dabigatran	Rivaroxaban
	N = 2153	N = 3089	N = 5286
Low‐dose aspirin	924 (43)	1349 (44)	2386 (45)
Clopidogrel	240 (11)	329 (11)	543 (10)
NSAIDs	216 (10)	343 (11)	695 (13)
Oral steroids	140 (7)	161 (5)	347 (7)
Antiarrhythmic drugs	323 (15)	533 (17)	932 (18)
Amiodarone	287 (13)	462 (15)	824 (16)
Βeta‐blockers	1258 (58)	1792 (58)	3177 (60)
ACE‐inhibitors	373 (17)	553 (18)	988 (19)
Diuretics	882 (41)	1230 (40)	2290 (43)
Statins	1193 (55)	1665 (54)	3020 (57)
Antidiabetic drugs	370 (17)	499 (16)	893 (17)
PPIs	950 (44)	1322 (43)	2388 (45)
H_2_RAs	170 (8)	306 (10)	521 (10)
Antidepressants	375 (17)	472 (15)	886 (17)
Polypharmacy^a^			
None	473 (22.0)	722 (23.4)	1007 (19.1)
1‐4	428 (19.9)	620 (20.1)	1077 (20.4)
5‐9	704 (32.7)	1016 (32.9)	1785 (33.8)
≥10	548 (25.5)	731 (23.7)	1417 (26.8)

*Note:* Data are n (%).

Abbreviations: ACE, angiotensin‐converting‐enzyme; H_2_RAs, H_2_‐receptor antagonists; NOAC, non‐vitamin K antagonist oral anticoagulant; NSAIDs, nonsteroidal anti‐inflammatory drugs; NVAF, nonsteroidal anti‐inflammatory drugs; PPIs, proton pump inhibitors.

^a^ Number of different medications in the previous 2 months.

### 3.2 Characteristics of NOAC use

Details about the index NOAC prescription are shown in Table 3. Rivaroxaban was mostly prescribed once daily (97.2%), which is the correct posology for stroke prevention in AF. The most frequent posology for apixaban and dabigatran was twice daily, this applied to 83.0% of patients prescribed apixaban and 82.5% of patients prescribed dabigatran. Among patients starting on apixaban, 38.0% were prescribed the standard 10 mg daily dose, over half were prescribed a reduced dose of 5 mg/day and 8.5% were prescribed a further reduced dose of 2.5 mg/day. For dabigatran, 30.9% of patients were prescribed the standard dose of 300 mg/day, while nearly a half of patients (49.5%) were prescribed a reduced dose of 220 mg/day, and the remaining patients (19.6%) received a dose of 150 mg/day or less. Among patients starting on rivaroxaban, 56.9% were prescribed the standard dose of 20 mg/day, while over a third (37.4%) received a reduced dose of 15 mg/day. Among new users of rivaroxaban there were 52 patients with an initial recorded dose of 40 mg/day. Of these, 9 (17.3%) were still prescribed rivaroxaban 3 months after starting therapy but not at a dose of 40 mg/day. Eight of the patients prescribed rivaroxaban 40 mg/day at the index date switched to another NOAC at 3 months. The mean age of all patients with severe renal impairment and prescribed a reduced dose NOAC was 82.6 years. Among anticoagulant naïve patients with at least 1 year of data following the index date, the percentage of patients with a first episode of continuous NOAC treatment of more than 180 days was as follows: apixaban, 44.6% of naïve patients vs 50.9% of non‐naïve patients; dabigatran, 51.4% of naïve vs 57.2% of non‐naïve patients; rivaroxaban, 52.7% of naïve patients vs 57.4% of non‐naïve patients. As shown in the Table S2, irrespective of the index NOAC, the majority of the NOAC prescriptions had either no gap between them (ie, they were immediately next to each other or overlapping prescriptions) or a small gap (0‐7 days) between them during follow‐up.

**TABLE 3:** Characteristics of the first NOAC prescription among patients with NVAF (July 2009 to June 2017)

Characteristic of index NOAC prescription	Apixaban	Dabigatran	Rivaroxaban
	N = 2153 n (%)	N = 3089 n (%)	N = 5286 n (%)
Posology			
Once daily	365 (17.0)	542 (17.5)	5140 (97.2)
Twice daily	1788 (83.0)	2547 (82.5)	146 (2.8)
Daily dose of first apixaban prescription (mg)			
2.5	183 (8.5)	—	—
5	1151 (53.5)	—	—
10	819 (38.0)	—	—
Daily dose of first dabigatran prescription (mg)			
75	—	23 (0.7)	—
110	—	330 (10.7)	—
150	—	253 (8.2)	—
220	—	1530 (49.5)	—
300	—	953 (30.9)	—
Daily dose of first rivaroxaban prescription (mg)			
2.5‐5	—	—	9 (0.2)
10	—	—	191 (3.6)
15	—	—	1978 (37.4)
20	—	—	3006 (56.9)
30	—	—	50 (0.9)
40	—	—	52 (1.0)
Duration of first continuous episode of NOAC use, days^a^ ^,^ ^b^			
1‐30	379 (28.5)	618 (25.1)	549 (14.4)
31‐90	157 (11.8)	249 (10.1)	704 (18.4)
91‐180	201 (15.1)	329 (13.4)	556 (14.5)
>180	594 (44.6)	1263 (51.4)	2016 (52.7)

Abbreviations: NOAC, non‐vitamin K antagonist oral anticoagulant; NSAIDs, nonsteroidal anti‐inflammatory drugs; NVAF, non‐steroidal anti‐inflammatory drugs.

^a^ Duration of first episode of continuous treatment (either no gap in treatment of >30 days between the end of the supply of a prescription and the start of the next prescription for the same NOAC or no further prescription).

^b^ Among patients with at least 1 year of follow‐up in the database (1331 for apixaban, 2459 for dabigatran, and 3825 for rivaroxaban).

## 4 DISCUSSION

Our study found that over the last decade, the use of NOACs among patients with NVAF and affiliated with a private healthcare regimen in Colombia, has markedly increased, with the observed time‐trends clearly reflecting the time different NOACs were introduced in the country. The decline in the incidence of patients with NVAF starting NOAC treatment on dabigatran—the first NOAC approved in Colombia for NVAF—was accompanied by an increase in the use of rivaroxaban and apixaban, which possibly reflects concerns about adverse gastrointestinal bleeding among prescribers although one study has reported that they have equivalent risks.^14^ Several studies in the US^15^, ^16^ and Europe^17^, ^18^, ^19^, ^20^, ^21^, ^22^, ^23^ have shown similar time‐trends in the use of these three NOACs in patients with NVAF. While the most recent data from our study (ie, for 2017) show that rivaroxaban was the most commonly prescribed NOAC in our Colombian study population, Zhu and colleagues^24^ found that apixaban was the predominantly prescribed NOAC in 2017 in their administrative database study of US NVAF patients, suggesting country‐specific differences in the contemporary use of specific NOACs.

The increasing use of NOACs in patients with NVAF in our Colombian study population, and in several others across the world,^15^, ^16^, ^17^, ^19^, ^20^, ^22^, ^23^, ^25^ suggests there has been a decline in warfarin use—the mainstay of oral anticoagulant (OAC) therapy in this patient population before the introduction of NOACs. These findings suggest that the benefits of NOACs over warfarin—both in terms of their favorable efficacy and safety profile^6^, ^7^, ^8^ as well as avoiding the need for regular clinic visits for coagulation monitoring ‐ is recognized by outpatient prescribers in Colombia. The preference for rivaroxaban could be due to its simple once‐a‐day administration, which would appeal to many prescribers in Colombia who recognize the importance of patient adherence to therapy to gain the full benefits of protection against ischaemic cerebrovascular events. The growing confidence in NOACs in our Colombian study population can also be inferred from our finding that approximately two‐thirds of the new users of NOACs in our study were OAC naïve. Also, approximately half of first‐time NOAC users still received treatment with their index NOAC after 6 months, indicating a certain level of adherence and tolerability among patients, providing a level of reassurance to physicians about prescribing this class of drugs. With respect to the initial NOAC dose prescribed in our patient population, less than 40% of patients starting NOAC therapy on apixaban, and less than a third of patients starting on dabigatran, received the standard daily dose, while 57% of patients starting on rivaroxaban were prescribed the standard daily dose. This shows that a large number of NOAC users with NVAF in our Colombian study population, especially those starting preventative treatment on dabigatran and apixaban, were prescribed a reduced dose. Studies among patients with NVAF in other countries have similarly shown the prescribing of reduced dose NOACs to be common,^26^, ^27^, ^28^ but that patients receiving a reduced dose do not always satisfy the dose reduction criteria on the drug label.^26^, ^29^, ^30^, ^31^, ^32^ Further investigation would be needed to see whether this is also the case in Colombia.

Demographic characteristics of our study population were broadly comparable to those in similar populations in other countries, with a mean age at first NOAC prescription a little over 75 years, and a slight predominance of men.^19^, ^29^, ^33^, ^34^ A high level of polypharmacy was apparent in our predominantly elderly study population, as also shown by Mueller and colleagues among NOAC users with NVAF in Scotland.^19^ As expected, and in line with other studies,^29^, ^33^, ^34^, ^35^, ^36^ hypertension—the predominant risk factor for AF—was the most common comorbidity among patients in our study, being recorded for about 80%. The presence of diabetes mellitus in about one‐fifth of patients in our study is also in line with findings among patients with NVAF in other countries,^29^, ^33^, ^34^, ^35^ although estimates have been slightly higher in some American cohorts.^29^, ^33^ Heart failure was present in about one‐third of patients with NVAF, which is both higher than some previous findings,^29^, ^33^, ^34^, ^35^ but lower than others,^23^ from studies across geographical locations.

The main strength of our study is the large population‐based database with detailed information on prescriptions. Also, the information recorded in the database enabled the characterization of patients in terms of demographics, comorbidities and comedications, which would not be present in other data sources based solely on prescribing records. Limitations of our study include the inability to characterize patients according to lifestyle factors such as smoking status, alcohol status and body mass index (from height and weight measurements) because these are not recorded in the database. Although we were able to identify patients with severe renal failure from ICD‐10 codes, we could not characterize patients in terms of mild or moderate renal failure because this information was not available in the database. Also, there may have been some under‐recording of some comorbidities. Given that the prescriptions in the database were those dispensed, persistence in the use of NOACs could be established, but treatment adherence was not analyzed because it was unknown whether patients actually took their medication. In addition, our results have limited generalizability because the patterns of NOAC use described in this study are those of patients in the contributory regime and not necessarily those of patients in the government subsidized regime.

## 5 CONCLUSION

We conclude that over the last decade, rivaroxaban has been the most commonly prescribed NOAC, followed by dabigatran, among patients with NVAF affiliated to a private health insurer in Colombia. Approximately half of patients continue to receive NOACs 6 months after the start of treatment, which suggests a certain level of adherence and tolerability, and a substantial percentage of patients, especially those starting therapy on apixaban, are prescribed a reduced dose. Studies are now needed focusing on the real‐world effectiveness and safety of NOACs in Colombia, as well as an evaluation of the appropriateness of reduced dosing.

## CONFLICT OF INTEREST

L. A. G. R. and A. R. work for CEIFE, which has received research funding from Bayer AG. L. A. G. R. also declares honoraria for serving on advisory boards for Bayer AG. J. E. M‐A. and A. G‐M. and M. E. M‐D. have received reimbursement from Bayer for international conference attendance. C. T‐Y. has no potential conflicts of interest to declare. Bayer AG had no role in the study design, the collection, analysis and interpretation of data, writing the report, and in the decision to submit the article for publication.

## ETHICS APPROVAL AND INFORMED CONSENT

The study protocol was approved by the bioethics committee of the Universidad Tecnológica de Pereira, Colombia, under the category of research without risk. In no case were personal data collected from patients.

## ETHICS STATEMENT

The study protocol was approved by the bioethics committee of the Universidad Tecnológica de Pereira, Colombia.

## Supporting information


**Table S1.** Comedications according to the ATC code among patients with nonvalvular atrial fibrillation (NVAF) and affiliated to a private healthcare regime prescribed a NOAC in Colombia

**Table S2.** Comedications according to the ATC code among patients with NVAF and affiliated to a private healthcare regime prescribed a NOAC in Colombia

## References

[1] January CT , Wann LS , Alpert JS , et al. AHA/ACC/HRS guideline for the management of patients with atrial fibrillation: a report of the American College of Cardiology/American Heart Association task force on practice guidelines and the Heart Rhythm Society. J Am Coll Cardiol. 2014;64(21):e1‐e76. 24685669 10.1016/j.jacc.2014.03.022

[2] Kirchhof P , Benussi S , Kotecha D , et al. ESC guidelines for the management of atrial fibrillation developed in collaboration with EACTS. Eur Heart J. 2016;37(38):2893‐2962. 27567408 10.1093/eurheartj/ehw210

[3] Wolf PA , Abbott RD , Kannel WB . Atrial fibrillation as an independent risk factor for stroke: the Framingham study. Stroke. 1991;22(8):983‐988. 1866765 10.1161/01.str.22.8.983

[4] Cubillos L , Haddad A , Kuznik A , Mould‐Quevedo J . Burden of disease from atrial fibrillation in adults from seven countries in Latin America. Int J Gen Med. 2014;7:441‐448. 25214802 10.2147/IJGM.S62819 PMC4159313

[5] Massaro AR , Lip GYH . Stroke prevention in atrial fibrillation: focus on Latin America. Arq Bras Cardiol. 2016;107(6):576‐589. 28558081 10.5935/abc.20160116 PMC5210462

[6] Connolly SJ , Ezekowitz MD , Yusuf S , et al. Dabigatran versus warfarin in patients with atrial fibrillation. N Engl J Med. 2009;361(12):1139‐1151. 19717844 10.1056/NEJMoa0905561

[7] Granger CB , Alexander JH , McMurray JJ , et al. Apixaban versus warfarin in patients with atrial fibrillation. N Engl J Med. 2011;365(11):981‐992. 21870978 10.1056/NEJMoa1107039

[8] Patel MR , Mahaffey KW , Garg J , et al. Rivaroxaban versus warfarin in nonvalvular atrial fibrillation. N Engl J Med. 2011;365(10):883‐891. 21830957 10.1056/NEJMoa1009638

[9] Gomez‐Outes A , Terleira‐Fernandez AI , Calvo‐Rojas G , et al. Direct oral anticoagulants for stroke prevention in patients with atrial fibrillation: meta‐analysis by geographic region with a focus on European patients. Br J Clin Pharmacol. 2016;82(3):633‐644. 27161800 10.1111/bcp.13005 PMC5338130

[10] Melgarejo‐Rojas E . Pharmacological prevention of embolism in atrial fibrillation and their risk scales for embolism and bleeding. Rev Colomb Cardiol. 2016;23:65–72.

[11] Machado‐Alba JE , Machado‐Duque ME , Gaviria‐Mendoza A . Time to modification of antidiabetic therapy in patients over the age of 65 years with newly diagnosed diabetes mellitus. Diabetes Res Clin Pract. 2020;162:108090. 32088311 10.1016/j.diabres.2020.108090

[12] Moreno‐Gutíerrez PA , Gaviria‐Mendoza A , Ochoa‐Orozco SA , Yepes‐Echeverri MC , Machado‐Alba JE . Long‐term users of benzodiazepines in Colombia: patterns of use and cessation of treatment. Drug Alcohol Depend. 2020;210:107962. 32220698 10.1016/j.drugalcdep.2020.107962

[13] Valladales‐Restrepo LF , Duran‐Lengua M , Castro‐Osorio EE , Machado‐Alba JE . Consistency between anticholinergic burden scales in the elderly with fractures. PLoS One. 2020;15(2):e0228532. 32092055 10.1371/journal.pone.0228532 PMC7039428

[14] Graham DJ , Reichman ME , Wernecke M , et al. Cardiovascular, bleeding, and mortality risks in elderly Medicare patients treated with dabigatran or warfarin for nonvalvular atrial fibrillation. Circulation. 2015;131(2):157‐164. 25359164 10.1161/CIRCULATIONAHA.114.012061

[15] Alalwan AA , Voils SA , Hartzema AG . Trends in utilization of warfarin and direct oral anticoagulants in older adult patients with atrial fibrillation. Am J Health Syst Pharm. 2017;74(16):1237‐1244. 28652320 10.2146/ajhp160756

[16] Barnes GD , Lucas E , Alexander GC , et al. National Trends in ambulatory Oral anticoagulant use. Am J Med. 2015;128(12):1300‐1305. e1302. 26144101 10.1016/j.amjmed.2015.05.044 PMC4658248

[17] Ruigomez A , Brobert G , Vora P , et al. Trends in use of rivaroxaban for prophylaxis and treatment in general practice in the United Kingdom between 2012 and 2015. Pharmacoepidemiology and Drug Safety. 2018;27(S2).

[18] Loo SY , Dell'Aniello S , Huiart L , et al. Trends in the prescription of novel oral anticoagulants in UKprimary care. Br J Clin Pharmacol. 2017;83(9):2096‐2106. 28390065 10.1111/bcp.13299 PMC5555878

[19] Mueller T , Alvarez‐Madrazo S , Robertson C , Bennie M . Use of direct oral anticoagulants in patients with atrial fibrillation in Scotland: applying a coherent framework to drug utilisation studies. Pharmacoepidemiol Drug Saf. 2017;26(11):1378‐1386. 28752670 10.1002/pds.4272 PMC5697642

[20] Bjerring Olesen JB , Sorensen R , Hansen ML , et al. Non‐vitamin K antagonist oral anticoagulation agents in anticoagulant naive atrial fibrillation patients: Danish nationwide descriptive data 2011‐2013. Europace. 2015;17(2):187‐193. 25236181 10.1093/europace/euu225

[21] Fay MR , Martins JL , Czekay B . Oral anticoagulant prescribing patterns for stroke prevention in atrial fibrillation among general practitioners and cardiologists in three European countries. European Heart Journal. 2016;37(Supplement 1):510.26726043

[22] Kjerpeseth LJ , Ellekjaer H , Selmer R , et al. Trends in use of warfarin and direct oral anticoagulants in atrial fibrillation in Norway, 2010 to 2015. Eur J Clin Pharmacol. 2017;73(11):1417‐1425. 28735494 10.1007/s00228-017-2296-1

[23] Ibanez L , Sabate M , Vidal X , et al. Incidence of direct Oral anticoagulant use in patients with non‐valvular atrial fibrillation and characteristics of users in six European countries (2008‐2015): a cross‐national drug utilization study. Br J Clin Pharmacol. 2019;73:1417–1425. 10.1111/bcp.14071 PMC6848911 31318059

[24] Zhu J , Alexander GC , Nazarian S , Segal JB , Wu AW . Trends and variation in Oral anticoagulant choice in patients with atrial fibrillation, 2010‐2017. Pharmacotherapy. 2018;38(9):907‐920. 29920705 10.1002/phar.2158 PMC6448138

[25] Camm AJ , Accetta G , Ambrosio G , et al. Evolving antithrombotic treatment patterns for patients with newly diagnosed atrial fibrillation. Heart. 2017;103(4):307‐314. 27647168 10.1136/heartjnl-2016-309832 PMC5293840

[26] Steinberg BA , Shrader P , Pieper K , et al. Frequency and outcomes of reduced dose non‐vitamin K antagonist anticoagulants: results from ORBIT‐AF II (the outcomes registry for better Informed treatment of atrial fibrillation II). J Am Heart Assoc. 2018;7(4):e007633. 29453305 10.1161/JAHA.117.007633 PMC5850192

[27] Staerk L , Gerds TA , Lip GYH , et al. Standard and reduced doses of dabigatran, rivaroxaban and apixaban for stroke prevention in atrial fibrillation: a nationwide cohort study. J Intern Med. 2018;283(1):45‐55. 28861925 10.1111/joim.12683

[28] Buchholz A , Ueberham L , Gorczynska K , et al. Initial apixaban dosing in patients with atrial fibrillation. Clin Cardiol. 2018;41(5):671‐676. 29542830 10.1002/clc.22949 PMC6489803

[29] Yao X , Shah ND , Sangaralingham LR , Gersh BJ , Noseworthy PA . Non‐vitamin K antagonist Oral anticoagulant dosing in patients with atrial fibrillation and renal dysfunction. J Am Coll Cardiol. 2017;69(23):2779‐2790. 28595692 10.1016/j.jacc.2017.03.600

[30] Barra ME , Fanikos J , Connors JM , Sylvester KW , Piazza G , Goldhaber SZ . Evaluation of dose‐reduced direct Oral anticoagulant therapy. Am J Med. 2016;129(11):1198‐1204. 27341955 10.1016/j.amjmed.2016.05.041

[31] Lavoie K , Turgeon MH , Brais C , et al. Inappropriate dosing of direct oral anticoagulants in patients with atrial fibrillation. J Atr Fibril. 2016;9(4):1478. 10.4022/jafib.1478 PMC5673313 29250254

[32] Pisters R , van Vugt SPG , Brouwer MA , et al. Real‐life use of rivaroxaban in The Netherlands: data from the Xarelto for prevention of stroke in patients with atrial fibrillation (XANTUS) registry. Neth Heart J. 2017;25(10):551‐558. 28674871 10.1007/s12471-017-1009-9 PMC5612862

[33] Lip GYH , Pan X , Kamble S , et al. Discontinuation risk comparison among 'real‐world' newly anticoagulated atrial fibrillation patients: Apixaban, warfarin, dabigatran, or rivaroxaban. PLoS One. 2018;13(4):e0195950. 29709012 10.1371/journal.pone.0195950 PMC5927458

[34] Vinogradova Y , Coupland C , Hill T , et al. Risks and benefits of direct oral anticoagulants versus warfarin in a real world setting: cohort study in primary care. BMJ. 2018;362:k2505. 29973392 10.1136/bmj.k2505 PMC6031213

[35] Forslund T , Wettermark B , Hjemdahl P . Comparison of treatment persistence with different oral anticoagulants in patients with atrial fibrillation. Eur J Clin Pharmacol. 2016;72(3):329‐338. 26613954 10.1007/s00228-015-1983-z

[36] García Rodríguez LA , Schink T , Bezemer ID , et al. High cardiovascular risk and frequent cardiovascular medication use among new users of rivaroxaban and vitamin K antagonists. Poster presentation at the American Heart Association Scientific Session 2018; November 10–14, 2018; Chicago, IL, USA.

