# Supplementary material for: Use of non‐vitamin K antagonist oral anticoagulants in Colombia: A descriptive study using a national administrative healthcare database
Source: Pharmacoepidemiol Drug Saf. 2020 Oct 15;30(4):426–34. doi: 10.1002/pds.5124 (PMC7983923; doi:10.1002/pds.5124)
Supplement: Supplementary file 1 — Table S1. Comedications according to the ATC code among patients with nonvalvular atrial fibrillation (NVAF) and affiliated to a private healthcare regime prescribed a NOAC in Colombia [file PDS-30-426-s001.docx]

**Supplementary table 1**. Comedications according to the ATC code among patients with NVAF and affiliated to a private healthcare regime prescribed a NOAC in Colombia.

| **Medications** | **ATC Code** | **Drug** |
| --- | --- | --- |
| ACE-inhibitors | C09AA01 | Captopril |
|  | C09AA02 | Enalapril |
|  | C09AA03 | Lisinopril |
|  | C09AA04 | Perindopril |
|  | C09AA05 | Ramipril |
|  | C09AA06 | Quinapril |
|  | C09AA07 | Benazepril |
|  | C09AA08 | Cilazapril |
|  | C09AA09 | Fosinopril |
|  | C09BA02 | Enalapril - combination |
|  | C09BA04 | Perindopril - combination |
|  | C09BA06 | Quinapril - combination |
|  | C09BA09 | Fosinopril - combination |
|  | C09BB02 | Enalapril - combination |
|  | C09BB04 | Perindopril - combination |
|  | C09BB10 | Trandolapril - combination |
| Acid-suppressive drugs/Antacids | A02AA04 | Magnesium hydroxide |
|  | A02AB01 | Aluminium hydroxide |
|  | A02AB10 | Aluminium compounds - combinations |
|  | A02AF02 | Ordinary salt combinations and antiflatulents (ie: with aluminium hydroxide) |
|  | A02AH | Antacids with sodium bicarbonate |
|  | A02BX02 | Sucralfate |
|  | A02BX05 | Bismuth subcitrate |
|  | A02BX13 | Alginic acid |
| Amiodarone | C01BD01 | Amiodarone |
| Antiarrhythmic drugs | C01BA01 | Quinidine |
|  | C01BC03 | Propafenone |
|  | C01BC04 | Flecainide |
|  | C01BD07 | Dronedarone |
|  | C07AA07 | Sotalol |
| Antidepressants | N06AA02 | Imipramine |
|  | N06AA04 | Clomipramine |
|  | N06AA06 | Trimipramine |
|  | N06AA09 | Amitriptyline |
|  | N06AA12 | Doxepin |
|  | N06AA15 | Butriptyline |
|  | N06AB03 | Fluoxetine |
|  | N06AB04 | Citalopram |
|  | N06AB05 | Paroxetine |
|  | N06AB06 | Sertraline |
|  | N06AB08 | Fluvoxamine |
|  | N06AB10 | Escitalopram |
|  | N06AX05 | Trazodone |
|  | N06AX11 | Mirtazapine |
|  | N06AX12 | Bupropion |
|  | N06AX16 | Venlafaxine |
|  | N06AX17 | Milnacipran |
|  | N06AX21 | Duloxetine |
|  | N06AX23 | Desvenlafaxine |
|  | N06CA01 | Amitriptyline and psycholeptics |
| Antidiabetic drugs | A10AB01 | Insulin (human) |
|  | A10AB04 | Insulin lispro |
|  | A10AB05 | Insulin aspart |
|  | A10AB06 | Insulin glulisine |
|  | A10AC01 | Insulin (human) - intermediate-acting |
|  | A10AD01 | Insulin (human) - intermediate-acting, combined with fast-acting |
|  | A10AD04 | Insulin lispro, combination |
|  | A10AD05 | Insulin aspart, combination |
|  | A10AE01 | Insulin (human) - long-acting |
|  | A10AE04 | Insulin glargine |
|  | A10AE05 | Insulin detemir |
|  | A10AE06 | Insulin degludec |
|  | A10BA02 | Metformin |
|  | A10BB01 | Glibenclamide |
|  | A10BB02 | Chlorpropamide |
|  | A10BB09 | Gliclazide |
|  | A10BB12 | Glimepiride |
|  | A10BD02 | Metformin and sulfonylureas - combinations |
|  | A10BD03 | Metformin and rosiglitazone |
|  | A10BD08 | Metformin and vildagliptin |
|  | A10BD10 | Metformin and saxagliptin |
|  | A10BD11 | Metformin and linagliptin |
|  | A10BD15 | Metformin and dapagliflozin |
|  | A10BD16 | Metformin and canagliflozin |
|  | A10BF01 | Acarbose |
|  | A10BG02 | Rosiglitazone |
|  | A10BG03 | Pioglitazone |
|  | A10BH01 | Sitagliptin |
|  | A10BH02 | Vildagliptin |
|  | A10BH03 | Saxagliptin |
|  | A10BH05 | Linagliptin |
|  | A10BJ01 | Exenatide |
|  | A10BJ02 | Liraglutide |
|  | A10BJ03 | Lixisenatide |
|  | A10BJ05 | Dulaglutide |
|  | A10BK01 | Dapagliflozin |
|  | A10BK02 | Canagliflozin |
|  | A10BK03 | Empagliflozin |
|  | A10BX02 | Repaglinide |
|  | A10BX03 | Nateglinide |
| Beta-blockers | C07AA05 | Propranolol |
|  | C07AA12 | Nadolol |
|  | C07AB02 | Metoprolol |
|  | C07AB03 | Atenolol |
|  | C07AB07 | Bisoprolol |
|  | C07AB12 | Nebivolol |
|  | C07AG02 | Carvedilol |
|  | C07BA05 | Propranolol and thiazides |
|  | C07BA12 | Nadolol and thiazides |
|  | C07BB02 | Metoprolol and thiazides |
|  | C07BB07 | Bisoprolol and thiazides |
|  | C07CB03 | Atenolol and thiazides |
| Clopidogrel | B01AC04 | Clopidogrel |
| Diuretics | C03AA03 | Hydrochlorothiazide |
|  | C03AX01 | Hydrochlorothiazide, combinations |
|  | C03BA11 | Indapamide |
|  | C03CA01 | Furosemide |
|  | C03CA02 | Bumetanide |
|  | C03CA04 | Torasemide |
|  | C03DA01 | Spironolactone |
|  | C03DA04 | Eplerenone |
|  | C03DB02 | Triamterene |
|  | C03EA01 | Hydrochlorothiazide and potassium-sparing agents |
|  | C03EB01 | Furosemide and potassium-sparing agents |
|  | C09DA01-8 | Angiotensin II receptor blockers (ARBs) and diuretics |
|  | C09DX01 | Valsartan, amlodipine and hydrochlorothiazide |
|  | C09XA52 | Aliskiren and hydrochlorothiazide |
| H_2_RAs | A02BA01 | Cimetidine |
|  | A02BA02 | Ranitidine |
|  | A02BA03 | Famotidine |
| Low-dose aspirin | B01AC06 | Acetylsalicylic acid |
| NSAIDs | M01AB01 | Indometacin |
|  | M01AB05 | Diclofenac |
|  | M01AB06 | Alclofenac |
|  | M01AB08 | Etodolac |
|  | M01AB11 | Acemetacini |
|  | M01AB16 | Aceclofenac |
|  | M01AC01 | Piroxicam |
|  | M01AC06 | Meloxicam |
|  | M01AE01 | Ibuprofen |
|  | M01AE02 | Naproxen |
|  | M01AE03 | Ketoprofen |
|  | M01AE04 | Fenoprofen |
|  | M01AE12 | Oxaprozin |
|  | M01AE51 | Ibuprofen, combinations |
|  | M01AH01 | Celecoxib |
|  | M01AH05 | Etoricoxib |
|  | M01AH06 | Lumiracoxib |
|  | M01AX17 | Nimesulide |
|  | M03BA53 | Methocarbamol, combinations excl. psycholeptics |
|  | N02BA01 | Acetylsalicylic acid |
|  | N02BA51 | Acetylsalicylic acid, combinations excl. psycholeptics |
|  | N02BA71 | Acetylsalicylic acid, combinations with psycholeptics |
|  | N02BE71 | Paracetamol, combinations with psycholeptics |
| Oral steroids | H02AA02 | Fludrocortisone |
|  | H02AB01 | Betamethasone |
|  | H02AB02 | Dexamethasone |
|  | H02AB04 | Methylprednisolone |
|  | H02AB06 | Prednisolone |
|  | H02AB07 | Prednisone |
|  | H02AB09 | Hydrocortisone |
|  | H02AB13 | Deflazacort |
| Other antihypertensives | C02AB01 | Methyldopa |
|  | C02AC01 | Clonidine |
|  | C02CA01 | Prazosin |
|  | C02CA04 | Doxazosin |
|  | C02DC01 | Minoxidil |
|  | C08CA01 | Amlodipine |
|  | C08CA03 | Isradipine |
|  | C08CA05 | Nifedipine |
|  | C08CA08 | Nitrendipine |
|  | C08CA09 | Lacidipine |
|  | C08DA01 | Verapamil |
|  | C08DB01 | Diltiazem |
|  | C09CA01 | Losartan |
|  | C09CA02 | Eprosartan |
|  | C09CA03 | Valsartan |
|  | C09CA04 | Irbesartan |
|  | C09CA06 | Candesartan |
|  | C09CA07 | Telmisartan |
|  | C09CA08 | Olmesartan medoxomil |
|  | C09DB01 | Valsartan and amlodipine |
|  | C09DB04 | Telmisartan and amlodipine |
|  | C09DB05 | Irbesartan and amlodipine |
|  | C09DB06 | Losartan and amlodipine |
|  | C09DX04 | Valsartan and sacubitril |
| Other antiplatelet | B01AC05 | Ticlopidine |
|  | B01AC07 | Dipyridamole |
|  | B01AC22 | Prasugrel |
|  | B01AC23 | Cilostazol |
|  | B01AC24 | Ticagrelor |
| PPIs | A02BC01 | Omeprazole |
|  | A02BC02 | Pantoprazole |
|  | A02BC03 | Lansoprazole |
|  | A02BC04 | Raveprazole |
|  | A02BC05 | Esomeprazole |
| Statins | C10AA01 | Simvastatin |
|  | C10AA02 | Lovastain |
|  | C10AA03 | Pravastatin |
|  | C10AA04 | Fluvastatin |
|  | C10AA05 | Atorvastatin |
|  | C10AA07 | Rosuvastatin |
|  | C10AA08 | Pitavastatin |
|  | C10BA02 | Simvastatin and ezetimibe |
|  | C10BA05 | Atorvastatin and ezetimibe |
|  | C10BA06 | Rosuvastatin and ezetimibe |
